# Hydroxylated Cinnamates Enhance Tomato Resilience to *Alternaria alternata*, the Causal Agent of Early Blight Disease, and Stimulate Growth and Yield Traits

**DOI:** 10.3390/plants12091775

**Published:** 2023-04-26

**Authors:** Yasser Nehela, Yasser S. A. Mazrou, Naglaa A. Taha, Abdelnaser A. Elzaawely, Tran Dang Xuan, Abeer H. Makhlouf, Asmaa El-Nagar

**Affiliations:** 1 Department of Agricultural Botany, Faculty of Agriculture, Tanta University, Tanta 31527, Egypt; 2 Business Administration Department, Community College, King Khalid University, Guraiger, Abha 62529, Saudi Arabia; 3 Department of Agriculture Economic, Faculty of Agriculture, Tanta University, Tanta 31511, Egypt; 4 Vegetable Diseases Research Department, Agricultural Research Center, Plant Pathology Research Institute, Giza 12619, Egypt; 5 Transdisciplinary Science and Engineering Program, Graduate School of Advanced Science and Engineering, Hiroshima University, Higashi-Hiroshima 739-8529, Japan; 6 Center for the Planetary Health and Innovation Science (PHIS), The IDEC Institute, Hiroshima University, Higashi-Hiroshima 739-8529, Japan; 7 Department of Agricultural Botany, Faculty of Agriculture, Minufiya University, Shibin El-Kom 32511, Egypt

**Keywords:** early blight, *Alternaria*, tomato, phenolic compounds, cinnamic acid, *ρ*-coumaric acid, caffeic acid, ferulic acid

## Abstract

The important vegetable crop, tomato, is challenged with numerous abiotic and biotic stressors, particularly the newly emerged fungicide-resistant strains of phytopathogenic fungi such as *Alternaria alternata*, the causal agent of early blight disease. The current study investigated the potential antifungal activity of four cinnamate derivatives including cinnamic acid, *ρ*-coumaric acid, caffeic acid, and ferulic acid against *A. alternata*. Our in vitro findings showed that all tested compounds exhibited dose-dependent fungistatic action against *A. alternata* when their concentrations were increased from 0.1, 0.3, 0.5, and 0.7, to 0.9 mM, respectively. The high concentration of ferulic acid (0.9 mM) completely inhibited the radial mycelial growth of *A. alternata* and it was comparable to the positive control (difenoconazole fungicide). Additionally, under greenhouse conditions, foliar application of the four tested cinnamates significantly reduced the severity of early blight disease without any phytotoxicity on treated tomato plants. Moreover, it significantly improved the growth traits (plant height, total leaf area, number of leaves per plant, and shoot fresh weight), total chlorophyll, and yield components (number of flowers per plant, number of fruits per plant, and fruit yield) of treated *A. alternata*-infected plants. Collectively, our findings suggest that cinnamate derivatives could be good candidates as eco-friendly alternatives to reduce the use of chemical fungicides against *A. alternata*.

## 1. Introduction

Tomato (*Solanum lycopersicum* L.) is one of the most important vegetable crops worldwide [1,2]. The fruits of tomatoes are highly nutrient-dense since they include high amounts of vitamins A and C as well as naturally occurring antioxidants [3]. Tomato is the second most significant horticultural crop [4]. In 2020, global fresh tomato production approached 185 million tons harvested from 5,167,388 hectares with an average yield of 36.89 tons per hectare [5]. It is worth mentioning that Egypt cultivates approximately 170,862 hectares of tomatoes annually with an average yield of 406,716 kg per hectare [5]. Tomato plants are susceptible to a wide range of phytopathogens including fungi, bacteria, viruses, and nematodes that attack tomatoes from seedlings to mature stages [6] causing several above- and below-ground diseases [7]. Fungal diseases, particularly those caused by ascomycetous fungi, are the most common tomato diseases [8].

Early blight, caused by two closely related ascomycetous species (*Alternaria solani* and *A. alternata*), is one of the most common tomato diseases [9,10,11]. Both *Alternaria* species can infect tomatoes, potatoes, peppers, and several plant species in the Solanaceae family [12,13,14]. Early blight disease destroys photosynthetic pigments, which significantly reduces growth and, as a result, productivity [13]. Additionally, it hinders the performance of photosystem II and reduces the amount of chlorophyll in infected leaves, slowing down photosynthesis [13,15,16]. Brown-to-dark-brown necrotic spots with concentric rings that appear on leaves, stems, and fruits are generally regarded as the disease’s symptoms [17,18].

Management of early blight disease on commercial farms mainly depends on disease monitoring, cultural control, planting resistant cultivars, and the application of synthetic agrochemicals such as fungicides. However, the intensive use of fungicides from the same chemical families leads to the development of fungicide-resistant strains that are insensitive to particular active compounds. Moreover, the widespread use of these fungicides has resulted in serious health and environmental issues, leading scientists all over the world to look for more sustainable, eco-friendly, and effective disease-management alternatives [19].

Phenolic compounds are a huge family of secondary metabolites with a wide variety of biological properties [20,21,22]. Although it has been demonstrated previously that several phenolic compounds exhibit antifungal activity against various phytopathogenic fungi, including *Fusarium graminearum* [23], *A. alternata* [24], *Penicillium expansum* [25], *A. solani* [1,26], *Plasmopara viticola* [27], and *Botrytis cinerea* [28], the potential antifungal activity of cinnamic acid and its derivatives is poorly studied. The most common straightforward derivatives of cinnamic acid are *ρ*-coumaric acid, caffeic acid, and ferulic acid [29]. Some antiviral, antibacterial, and antifungal activities of cinnamic acid and its derivatives, whether obtained from plant materials or synthetically produced, have been reported previously [30]. For instance, cinnamic acid and its derivatives showed antifungal efficacy against ascomycetes fungi such as *Cochliobolus lunatus*, *Aspergillus niger*, and the basidiomycetes fungus *Pleurotus ostreatus* [31]. Moreover, cinnamic acid has antifungal action against *Sclerotinia sclerotiorum* the causal agent of Sclerotinia stem [32]. Although cinnamic acid and its derivatives have a wide range of applications, nothing is known about their antifungal effectiveness against *A. alternata*.

In the current study, we investigated the antifungal activity of four phenolic compounds including cinnamic acid, *ρ*-coumaric acid, caffeic acid, and ferulic acid against *A. alternata* both in vitro and in vivo. Additionally, we studied their potential effect(s) on the growth and yield components of *A. alternata*-infected tomato plants under greenhouse conditions.

## 2. Results

### 2.1. Pathogenicity Test

**Figure 1:**
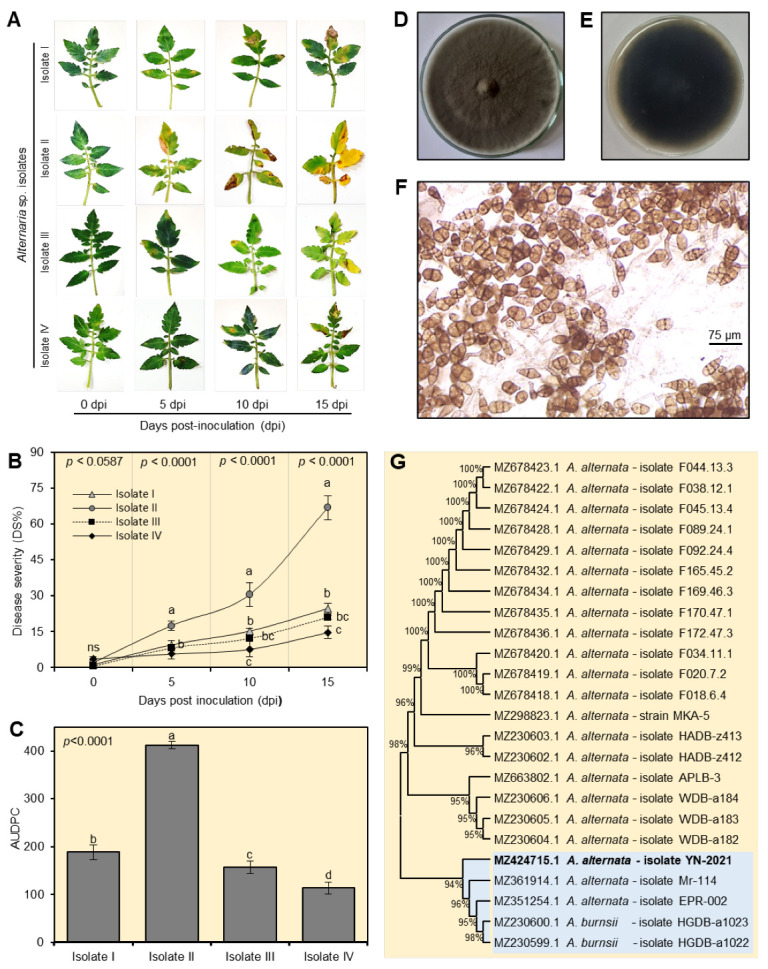
Pathogenicity and identification of *Alternaria alternata* isolate. (**A**) Development of early blight symptoms on tomato leaves (cv. Super strain B-F1 hybrid) after infection with four different *A. alternata* isolates at 0-, 5-, 10-, and 15-days post inoculation (dpi). (**B**) Disease severity (%) of four *A. alternata* isolates on tomato leaves (cv. Super strain B-F1 hybrid) under greenhouse conditions. (**C**) Area under disease progress curve (AUDPC) of four *A. alternata* isolates on tomato leaves (cv. Super strain B-F1 hybrid) under greenhouse conditions. In panels (**B**,**C**), values represent six biological replicates’ means ± standard deviations (mean ± SD; *n* = 6). Different letters denote significant differences according to Tukey’s HSD test (*p* ≤ 0.05). (**D**,**E**) Morphological characterization of *A. alternata* colony cultured on PDA from the top and the bottom of the Petri dish, respectively, after 7 days of incubation at 27 ± 1 °C. (**F**) Microscopic examination of *A. alternata* conidia. (**G**) Maximum-likelihood phylogenetic tree using ITS-5.8S rDNA sequence of *A. alternata* isolate YN-2021 (GenBank Accession No. MZ424715.1) (highlighted in bold).

Four isolates of *A. alternata* were isolated from tomato materials exhibiting typical symptoms of early blight disease. The pathogenicity of the four isolates was examined under greenhouse conditions. Generally, all isolates were pathogenic and produced typical early blight symptoms (Figure 1A). However, isolate II was the most aggressive one and caused the most severe disease severity on tomato leaves at 5, 10, and 15 dpi (17.55 ± 2.08, 30.50 ± 4.81, and 66.75 ± 5.112%; Figure 1B), as well as the highest AUDPC (411.87 ± 7.73; Figure 1C).

### 2.2. Morphological and Molecular Identification of Alternaria alternata

Isolate II showed typical morphological (Figure 1D,E) and microscopic characteristics (Figure 1F) with *Alternaria* species. The mycelial growth on the PDA medium was olivaceous, gray, dark gray to black with woolly colonies (Figure 1D) and the bottom side was almost black (Figure 1E). Light microscopy examination revealed that conidiophores are erect, septate, and geniculate. The conidia are borne singly or in chains. The conidia are brownish, obclavate, and smooth-walled with a short conical or cylindrical beak (Figure 1F) which are identical to those in the genus *Alternaria.* The most aggressive isolate (isolate II) was selected for further genetic identification at the species level. Briefly, the evolutionary analysis showed that the query sequence had a high similarity with *A. alternata* (Figure 1G). The new sequence was deposited to the NCBI database named “*A. alternata* isolate YN-2021” (GenBank Accession No. MZ424715.1).

### 2.3. In Vitro Antifungal Activity of Cinnamates against A. alternata

**Figure 2:**
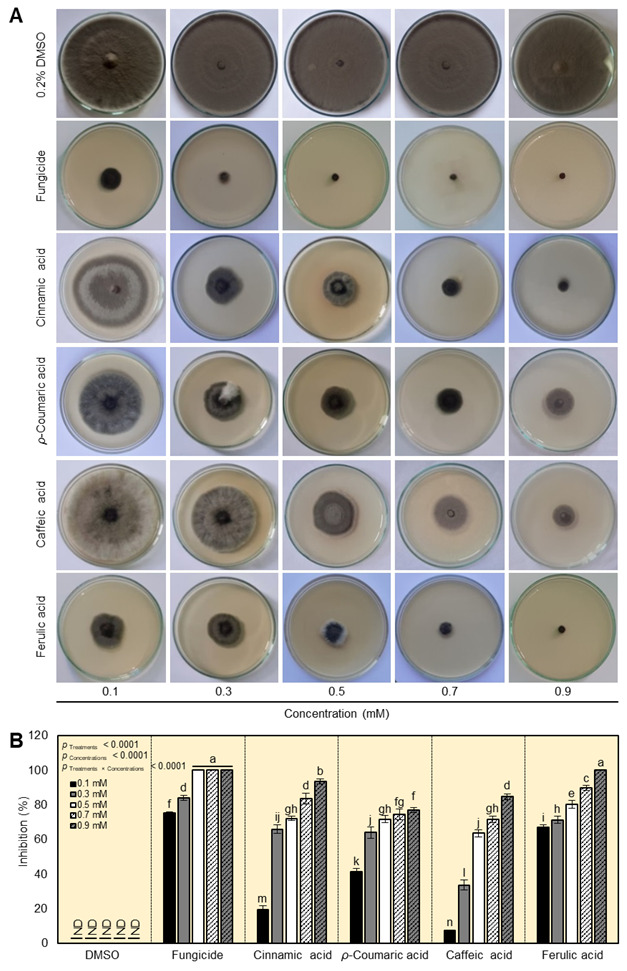
In vitro antifungal activity of four cinnamates (cinnamic acid, *ρ*-coumaric acid, caffeic acid, and ferulic acid) against *Alternaria alternata*. (**A**) Inhibition of the mycelial radial growth of *A. alternata* on PDA media after the treatment with one of five final concentrations (0.1, 0.3, 0.5, 0.7, and 0.9 mM) of four cinnamate derivatives. (**B**) Inhibition (%) of mycelial radial growth of *A. alternata* after treatment with one of five final concentrations (0.1, 0.3, 0.5, 0.7, and 0.9 mM) of four cinnamate derivatives. Values represent six biological replicates’ means ± standard deviations (mean ± SD; *n* = 6). Different letters denote significant differences according to Tukey’s HSD test based on the *p*-value of the interaction (*p* _Treatment×Concentrations_ ≤ 0.05). ND: not detected.

In vitro, the antifungal activity of four cinnamates (cinnamic acid, *ρ*-coumaric acid, caffeic acid, and ferulic) against *A. alternata* was tested. Generally, all tested cinnamates exposed dose-dependent antifungal activity against *A. alternata* and notably inhibited the mycelial radial growth on PDA media (Figure 2A). Ferulic acid was the most effective cinnamate and completely inhibited (100%) the mycelial growth at its highest concentration (0.9 mM) and it was comparable with the commercial fungicide Score (positive control), followed by cinnamic acid (93.62 ± 1.33%) at the same concentration (0.9 mM) (Figure 2B).

**Figure 3:**
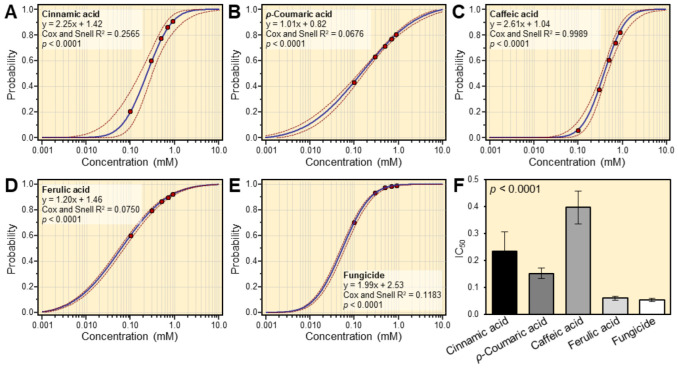
Probit modeling of the fungistatic effects of four cinnamates against *A. alternata*: (**A**) cinnamic acid, (**B**) *ρ*-coumaric acid, (**C**) caffeic acid, (**D**) ferulic acid, and (**E**) commercial fungicide Score, 25% (difenoconazole 25 EC; positive control). Red dots represent six biological replicates’ means ± standard deviations (mean ± SD; *n* = 6), whereas blue solid lines represent the dose–response regression lines. The estimated regression lines’ 95% confidence intervals (CI) are edged with dotted red lines. (**F**) The half-maximal inhibitory concentration (IC_50_) values (mM) of four tested cinnamates and the fungicide difenoconazole against *A. alternata*.

The probit regression analyses (Figure 3) showed that cinnamic acid (Figure 3A), *ρ*-coumaric acid (Figure 3B), caffeic acid (Figure 3C), ferulic acid (Figure 3D), and fungicide (Figure 3E), had a similar tendency in terms of slope values. In addition, ferulic acid (y = 1.20x + 1.46, *p* < 0.0001) showed better antifungal activity against *A. alternata* compared with other cinnamates. Additionally, both the IC_50_ and IC_99_ of the four tested cinnamates are listed in Table 1. Ferulic acid (IC_50_ = 0.06 mM) was found to have the lowest IC_50_ (Table 1 and Figure 3F) as well as the lowest IC_99_ (Table 1). Nevertheless, caffeic acid had the highest IC_50_, followed by cinnamic acid (Table 1).

### 2.4. Cinnamate Derivatives Reduce the Severity of Tomato Early Blight

**Figure 4:**
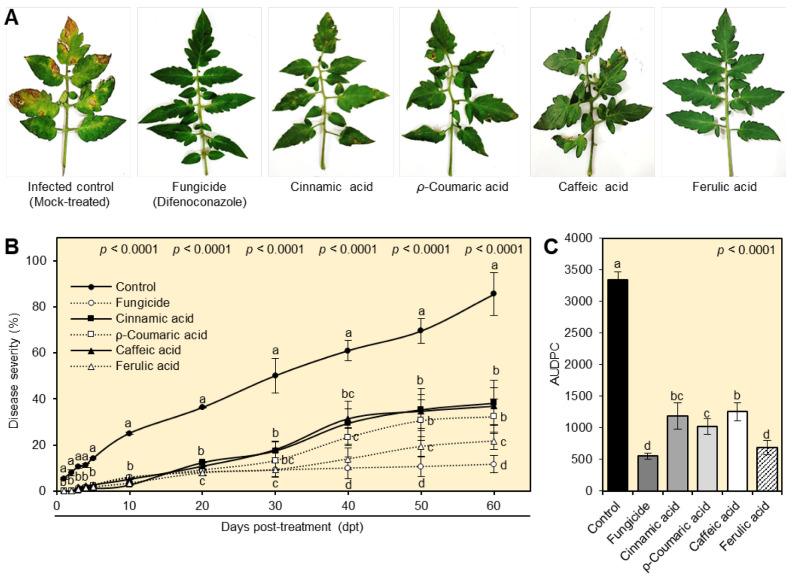
Effect of four tested cinnamates on evaluation of tomatoes with early blight disease caused by *A. alternata* under greenhouse conditions. (**A**) Symptoms of early blight disease caused by *A. alternata* on tomato leaves after the treatment with four cinnamates. (**B**) Severity (%) of early blight disease on tomato leaves with a 10-day interval and until 60 days post treatment (dpt) with four different cinnamates under greenhouse conditions. (**C**) The area under disease progress curve (AUDPC) of tomatoes with early blight disease under greenhouse conditions. Values represent six biological replicates’ means ± standard deviations (mean ± SD; *n* = 6). Different letters denote significant differences according to Tukey’s HSD test (*p* ≤ 0.05).

In comparison to untreated infected plants, foliar applications of cinnamate derivatives mitigated the severity of tomato early blight disease under greenhouse conditions (Figure 4A). All studied phenolic compounds considerably decreased the percentage of disease severity without significant differences between them at the first seven days post treatment (dpt). Nevertheless, at 10, 20, 30, 40, 50, and 60 dpt, respectively, ferulic acid was the most efficient compound and had the lowest disease severity (%) throughout the experiment (3.42 ± 0.04, 7.89 ± 1.18, 9.31 ± 3.09, 13.96 ± 4.63, 19.4 ± 4.22, and 21.76 ± 3.71% at 10, 20, 30, 40, 50, and 60 dpt, respectively). Additionally, foliar application of cinnamic acid, *ρ*-coumaric acid, caffeic acid, or ferulic acid dramatically lowered the AUDPC compared with the non-treated control (Figure 4B). On the other hand, ferulic acid had the lowest AUDPC value (686.28 ± 110.08), with no significant differences from the positive control, while the mock-treated control recorded the highest values (3337.95 ± 130.28). All noteworthy, these results show that the exogenous application of cinnamic acid and its derivatives reduces the negative effects of *A. alternata* on tomato leaves and inhibits the development of disease symptoms.

### 2.5. Cinnamates Enhance the Growth Characteristics of A. alternata-Infected Tomato Plants

**Figure 5:**
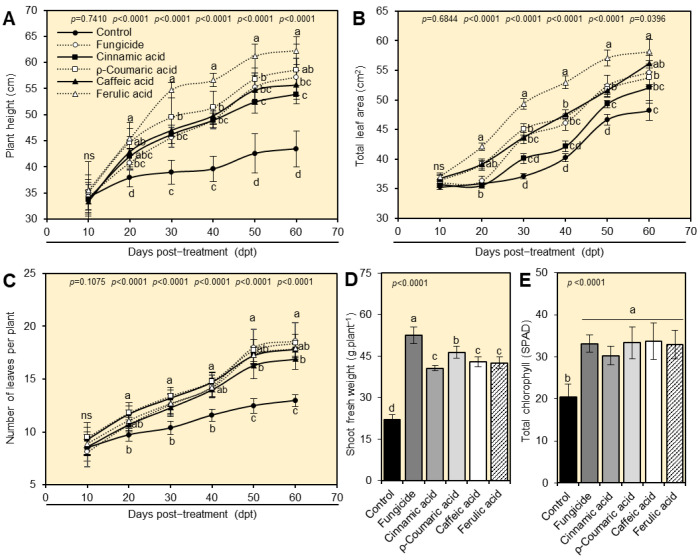
Effect of four tested cinnamates (cinnamic acid, *ρ*-coumaric acid, caffeic acid, and ferulic acid) on the growth of tomato plants (cv. Super strain B-F1 hybrid) affected by early blight disease caused by *Alternaria alternata* under greenhouse conditions: (**A**) plant height (cm), (**B**) total leaf area (cm^2^), and (**C**) number of leaves per plant at 0, 10, 20, 30, 40, 50, and 60 days post treatment (dpt) with four different cinnamates under greenhouse conditions. (**D**) Shoot fresh weight (g.plant^−1^), and (**E**) total chlorophyll (SPAD) at 60 dpt with four different cinnamates under greenhouse conditions. Values represent six biological replicates’ means ± standard deviations (mean ± SD; *n* = 6). Different letters denote significant differences according to Tukey’s HSD test (*p* ≤ 0.05).

Foliar application of cinnamic, *ρ*-coumaric, caffeic, and ferulic acids significantly enhanced plant height (Figure 5A), total leaf area (Figure 5B), and the total number of leaves per plant (Figure 5C) throughout 60 dpt. Ferulic acid had the highest effect on most vegetative parameters of tomato plants. Additionally, the exogenous application of cinnamate derivatives dramatically enhanced shoot fresh weight per plant, as well as the total chlorophyll when compared with mock-treated control plants (Figure 5D,E). The tomato plants treated with *ρ*-coumaric acid had the highest fresh weight, followed by ferulic, cinnamic, and caffeic acids, all of which were significantly higher than the mock-treated control. It is worth mentioning that all tested treatments significantly enhanced the total chlorophyll in tomato-infected leaves with no significant differences between them (Figure 5E).

### 2.6. Cinnamate Derivatives Increase Yield Components of A. alternata-Infected Tomato Plants

**Figure 6:**
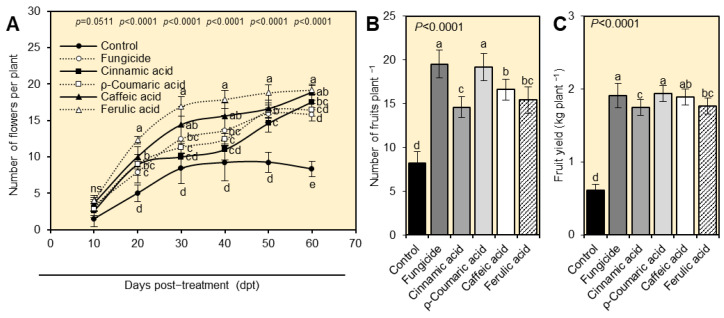
Effect of four tested cinnamates (cinnamic acid, *ρ*-coumaric acid, caffeic acid, and ferulic acid) on the yield components of tomato plants (cv. Super strain B-F1 hybrid) affected by early blight disease caused by *Alternaria alternata* under greenhouse conditions. (**A**) Number of flowers per plant at 10-, 20-, 30-, 40-, 50-, and 60-days post-treatment (dpt) with four different cinnamates under greenhouse conditions. (**B**) The number of fruits plant^−1^, and (**C**) fruit yield (kg plant^−1^) at the end of the experiment after foliar application of four different cinnamates under greenhouse conditions. Values represent six biological replicates’ means ± standard deviations (mean ± SD; *n* = 6). Different letters denote significant differences according to Tukey’s HSD test (*p* ≤ 0.05).

In comparison with mock-treated control, foliar application of cinnamate derivatives significantly increased all yield attributes of *A. alternata*-infected tomato plants, including the total number of flowers per plant (Figure 6A), the number of fruits per plant (Figure 6B), and the total fruit yield per plant (Figure 6C). Ferulic acid-treated plants had the highest number of flowers per plant (19.20 ± 0.61 flowers per plant at 60 dpt). However, *ρ*-coumaric-treated plants had the highest number of fruits (19.20 ± 1.55 fruits plant^−1^), and fruit yield (1.94 ± 0.11 kg plant^−1^) without significant differences from the fungicide-treated plants (19.51 ± 1.58 fruits plant^−1^ and 1.91 ± 0.11 kg plant^−1^, respectively).

## 3. Discussion

Chemical control of early blight disease using fungicides is still the main disease management strategy because of the absence of resistant cultivars. However, the extensive use of agrochemicals, such as fungicides, increases the selective pressure which speeds up the evolution of fungicide-resistant fungal strains. Therefore, it is necessary to search for novel eco-friendly alternatives with a unique mechanism of action against *Alternaria alternata*. Interestingly, phenolic compounds—the most prevalent class of plant-based natural products—exhibit a variety of biological characteristics, including antifungal action [33].

In general, phenolic compounds, and derivatives of cinnamic acid in particular, showed potent antifungal activity against several phytopathogenic fungi, including *Botrytis cinerea* [28], *Aspergillus niger* [31], *Sclerotinia sclerotiorum* [32], *A. solani* [1,26], and the oomycetes *Plasmopara viticola* [27]. For example, previously, we have shown that gallic acid and two of its derivatives (syringic acid and pyrogallic acid) might be a sustainable, eco-friendly control strategy against *A. solani* [1]. Likewise, benzoic acid and two of its hydroxylated derivatives, *ρ*-hydroxybenzoic acid and protocatechuic acid, significantly suppress the early blight of tomato caused by *A. solani* [26].

In the current study, we followed up our previous studies with an investigation of the potential antifungal activity of four cinnamate derivatives including cinnamic acid, *ρ*-coumaric acid, caffeic acid, and ferulic acid against *A. alternata.* Cinnamate derivatives are naturally occurring organic compounds that are found in plants and have a broad variety of biological functions including antibacterial, antiviral, and antifungal activity [30]. However, our knowledge about the potential biological role(s) of cinnamic acid and its derivatives against early blight disease is still limited.

Cinnamate derivatives alleviate the negative effects of phytopathogenic fungi on infected plants via a complex multilayered defense system that includes several biochemical and molecular mechanisms. The first mechanism proposed that cinnamate derivatives have strong antifungal activity against phytopathogens [1,26]. Our in vitro studies proved that the four tested cinnamate derivatives exhibited potent dose-dependent fungistatic action against *A. alternata*. The mycelial development of *A. alternata* was effectively reduced by the high concentrations (0.9 mM) of all tested cinnamates, particularly ferulic acid, and it was comparable to the positive control (difenoconazole fungicide), with no significant difference between them. The antifungal activity of cinnamate derivatives is linked with their structural characteristics [34,35]. For instance, phenyl ring replacement enhances the antifungal properties of cinnamates, and the nature and position of the substituent groups have generally been connected to the biological activity of different cinnamates [35].

In the current study, the hydroxylation of cinnamic acid by adding a hydroxyl group to the *ortho* and/or *meta* positions to form *ρ*-coumaric acid (also known as 4-hydroxycinnamic acid; the hydroxyl group was added to the *meta* position) or to form caffeic acid (also known as 3,4-dihydroxycinnamic acid; two hydroxyl groups were added to the *ortho* and *meta* positions, respectively) did not significantly enhance their fungal activity against *A. alternata*. However, both hydroxylation and methylation of cinnamic acid by adding two hydroxyl groups to the *ortho* and *meta* positions and adding a methyl group to the *ortho* position to form ferulic acid (also known as 3-methoxy-4-hydroxycinnamic acid) significantly enhanced its antifungal activity. Ferulic acid was the most potent compound among the tested compounds, and this may be due to the presence of the electron-withdrawing CH_3_ group where the presence of electron-withdrawing groups on the phenyl ring contributed to the antifungal activity [31]. However, more research is necessary to fully comprehend the molecular and cellular mechanisms behind the antifungal effects of cinnamic acid and its derivatives.

Another mechanism of the antifungal activity of cinnamates is due to their interaction with the fungal cytochrome P450 (CYP) enzyme family. The host plants produce several chemical compounds such as terpenes, phenolic compounds, and polycosanols, to defend themselves against various fungal pathogens. In response, fungi have evolved mechanisms to dispose of these toxic chemicals or convert them enzymatically [31]. CYP enzymes play a vital role in versatile metabolism and fungal adaptation to ecological niches [36,37], as well as the detoxification of plant-derived toxins [31]. For example, the majority of traditional antifungal treatment still relies on azole inhibitors that target the enzyme sterol 14α-demethylase (CYP51), which is involved in the demethylation of lanosterol, a precursor to ergosterol in the fungal membranes [34]. It is worth mentioning that cinnamate derivatives are involved in the inhibition of benzoate 4-hydroxylase (CYP53), which is unique and functionally described in ascomycetes [31]. Collectively, we suggest that cinnamic acid and its derivatives inhibited the growth of *A. alternata* by negatively affecting the CYP enzymes.

Furthermore, our greenhouse studies demonstrated that none of the four tested cinnamates caused any phytotoxicity in treated tomato plants while considerably reducing the disease severity (%) and area under the disease progress curve (AUDPC) of early blight disease. These results are consistent with our earlier research, which showed that tomato plants treated with exogenous applications of gallic acid and its derivatives or benzoic acid and its derivatives effectively slowed the onset of disease symptoms and reduced the AUDPC [1,26]. Likewise, the exogenous application of cinnamic acid significantly enhanced the heat tolerance of cucumber seedlings and improved growth parameters when used at 50 μM [38]. Similarly, the treatment of rice plants infected with *Rhizoctonia solani* with some phenolic compounds led to reduced disease and improved growth and productivity of rice plants [39]. Our findings suggest that the exogenous application of cinnamic acid and its derivatives slows the spread of early blight disease and mitigates the harmful effects of *A. alternata* on tomato leaves. The physiological and pharmacological mechanisms underpinning these roles, however, are poorly understood.

Additionally, the exogenous application of cinnamic acid and its derivatives enhances the growth of tomato plants infected with *A. alternata* (total chlorophyll, number of leaves, and plant height), as well as the yield components (number of flowers per plant, number of fruits per plant, and fruit yield). Cinnamic acid has also been utilized for a very long time as a raw material for preservatives for fruits and vegetables. It can greatly increase root activity and improve plant resistance [40]. However, further research is needed to fully understand the beneficial effects of cinnamic acid and its derivatives on the treated plants, albeit it may be because the severity of the disease has lessened.

## 4. Materials and Methods

### 4.1. Tested Compounds

**Figure 7:**
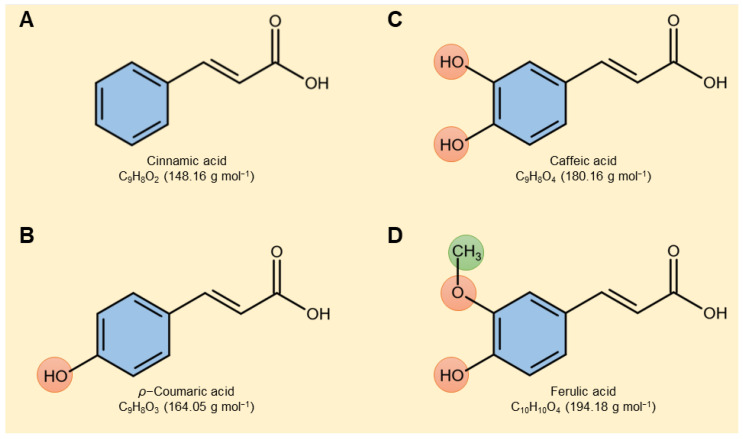
Chemical structures of four tested cinnamates. (**A**) Cinnamic acid, (**B**) *ρ*-coumaric acid, (**C**) caffeic acid, and (**D**) ferulic acid. the molar mass of each compound (g·mol^−1^) is mentioned between parentheses under its chemical formula.

Cinnamic acid (Figure 7A) and three of its derivatives including *ρ*-coumaric acid (Figure 7B), caffeic acid (Figure 7C), and ferulic acid (Figure 7D) were obtained from Sigma-Aldrich (Sigma-Aldrich Chemie GmbH-Schnelldorf, Schnelldorf, Germany). Each compound was dissolved in 2 mL of dimethyl sulfoxide (DMSO) and then adjusted to a final volume of 100 mL using sterilized water to prepare a 5 mM stock solution. In all subsequent experiments, this stock solution was diluted with distilled water right before utilization.

### 4.2. Plant Materials

Tomato (*Solanum lycopersicum* Mill. cv. Super strain B-F1 hybrid) seedlings were obtained from the Department of Vegetable Diseases (DVD), Institute of Plant Pathology Research (IPPR), Agricultural Research Center (ARC), Egypt.

### 4.3. Pathogen Isolation and Identification

Four isolates of *A. alternata* were isolated from tomato fruits exhibiting typical symptoms of early blight disease using the direct transfer and hyphal tip techniques. Briefly, infected tomato fruits were collected from a local market, then the leathery black spots, with raised concentric ridges, were cut into small pieces, cleaned with distilled water, and disinfected with sodium hypochlorite (2%) for 3 min to sterilize the surface of diseased tissue. After sterilizing the surface, it was rinsed three times with sterile distilled water before allowing it to air dry. Subsequently, the sterilized diseased tissue was transferred using a sterile needle to potato dextrose agar (PDA) plates containing 0.5 g L^−1^ of streptomycin to prevent bacterial contamination. The loaded plates were incubated for 7 days at 25 ± 2 °C. Later, all isolates were purified using the single-spore technique, subcultured at 45-day intervals, and maintained on PDA for further studies. The purified isolates were firstly characterized based on their cultural, morphological, and macroscopic characteristics, then molecularly identified based on the sequence of their internal transcribed spacer (ITS) region [41].

### 4.4. Pathogenicity Test

The pathogenicity of purified isolates was tested under greenhouse conditions using 30-day-old tomato plants. Briefly, conidial suspensions (with a concentration of 1 × 10^6^ conidia mL^−1^) of the four *A. alternata* isolates were separately sprayed on test plants. To help the spores adhere to plant tissues, 0.05% Tween 20 was added to the spore suspensions. The control plants were sprayed with sterile distilled water that had been mixed with 0.05% Tween 20. Inoculated plants were covered with plastic bags for two days. Disease symptoms comparable to those seen on the original plant materials began to appear five days post-inoculation (dpi). No symptoms of leaf blight appeared on control plants. The disease severity was assessed using a grading system suggested by Pandey, et al. [42]. The disease index (DI) was calculated for each isolate as well as the area under the disease progression curve (AUDPC) according to the equation given by Jeger and Viljanen-Rollinson [43] and Haynes and Weingartner [44], respectively.

### 4.5. Molecular Identification

For molecular identification, the fungal mycelium of the most aggressive isolate grown on sterile potato dextrose broth was harvested using cheesecloth, cleaned twice using sterile water, and air-dried. Subsequently, genomic DNA was extracted from approximately 100 mg fungal mycelium after grinding to a fine powder with liquid nitrogen as described by Atallah and Yassin [45], and Atallah, et al. [46], and purified, and the targeted ITS region sequences were PCR-amplified, purified, then sequenced using the Sanger method. Consensus sequences were assembled, compared with the most recent data in GenBank, then added to the GenBank database at the NCBI.

### 4.6. Antifungal Activity

The agar diffusion method was used to evaluate the in vitro antifungal activity of four tested cinnamates (cinnamic acid, *ρ*-coumaric acid, caffeic acid, and ferulic acid) [47]. A proper concentration of each compound, as well as DMSO (negative control) and the commercial fungicide Score, 25% (difenoconazole 25 EC; positive control), was mixed with 20 mL of the PDA medium in a sterile Petri dish to make gradient serial dilutions of five final concentrations (0.1, 0.3, 0.5, 0.7, and 0.9 mM). After media solidification, a 5 mm-diameter mycelial plug of the most aggressive *A. alternata* isolate was transferred to the Petri dishes, and the fungal development was observed after incubation at 25 ± °C for 7 days or until the mycelial growth covered the control plate. The experiment was repeated three times with six biological replicates per treatment each time. The percentage of growth inhibition was calculated using Equation (1):

(1) $$\mathrm{Inhibition}\;(\%)\;=\;\frac{A-B}{A}\times 100$$

where “A” denotes mycelial growth in the control and “B” denotes mycelial growth in the treatment. The whole experiment was repeated twice with six biological replicates per treatment.

Moreover, probit regression analysis was used to fit the probit/logit sigmoid dose–response curves and calculate half-maximal inhibitory concentration (IC_50_) and the inhibitory concentration (IC_99_) with 95% confidence intervals [48].

### 4.7. Greenhouse Experiments

30-day-old seedlings of early blight-susceptible tomato cultivar (Super strain B-F1 hybrid) were transplanted into plastic pots (30 cm in diameter) filled with sterilized clay soil and maintained under greenhouse conditions (25 ± 1 °C, 80 ± 2% RH, and 8:16-h light: dark photocycle) at DVD-IPPR/ARC, Sakha Agricultural Research Station, Kafr El-Sheikh, Egypt. During the whole experiment, pots were watered once a week, and all other standard horticultural practices and fertilization were performed as recommended. Two weeks after transplanting, infection with *A. alternata* and treatment with cinnamate derivatives were carried out as follows.

#### 4.7.1. Infection with *A. alternata*

Tomato seedlings were sprayed using a manual one-gallon atomizer, two weeks after transplantation with a conidial suspension of a 10-day-old culture of *A. alternata* (1 × 10^6^ conidia mL^−1^ produced in sterile water).

#### 4.7.2. Treatment with Cinnamate Derivatives

Infected tomato plants were sprayed with approximately 30 mL plant^−1^ of 0.9 mM solution of one of the investigated phenolic compounds (cinnamic acid, *ρ*-coumaric acid, caffeic acid, and ferulic acid) at 24 h post inoculation (hpi). Sterilized 0.2% DMSO was used as a negative control, whereas the recommended dose of the commercial fungicide Score was used as a positive control. Throughout the season, the four cinnamates were applied topically four times, with a 10-day interval between applications. The entire experiment was repeated twice under greenhouse conditions.

#### 4.7.3. Disease Assessments

The early blight disease severity was assessed every day during the first 7 days post treatment (dpt) then every 10 days till 60 dpt using the method established by Pandey, et al. [42] as mentioned above. Moreover, the AUDPC was calculated using the equation given by Jeger and Viljanen-Rollinson [43] and Haynes and Weingartner [44], respectively.

#### 4.7.4. Assessment of Vegetative Growth and Yield Components

Plant height (cm), total leaf area (cm^2^), and number of leaves per plant were recorded at 10, 20, 30, 40, 50, and 60 dpt, whereas shoot fresh weight (g plant^−1^), as well as total chlorophyll (SPAD) was measured once at 60 dpt. Likewise, the number of flowers per plant was assessed at 10, 20, 30, 40, 50, and 60 dpt as a part of yield components, whereas the number of fruits per plant and fruit yield (kg plant^−1^) was recorded at the end of the experiment.

### 4.8. Experimental Design and Statistical Analysis

In vitro antifungal activity of four tested cinnamates was carried out using a split-plot design using the compounds (treatments) in the main plots and the concentrations in the sub-plots. The analysis of variance (ANOVA) was used to check significant differences for the main effects of tested cinnamates (*p*_Treatments_), concentrations (*p*_Concentrations_), and their interaction (*p*_Treatment×Concentrations_). The Tukey’s honestly significant difference (HSD) test was used for post hoc analysis based on the *p*-value of the interaction (*p*_Treatment×Concentrations_ ≤ 0.05). Otherwise, all experiments were carried out throughout the study using a completely randomized design (CRD). All data were statistically analyzed using ANOVA and the HSD test (*p* ≤ 0.05) for post hoc analysis and pairwise comparisons.

Throughout the study, all experiments were carried out using six biological and two technical replicates per treatment (*n* = 6). Technical replicates were used to test the reproducibility within the same experiment. Moreover, all experiments were repeated independently to confirm the reproducibility of all experimental observations. However, because our findings showed high reproducibility and the values of each pair of technical replicates were consistent and very close to each other, technical replicates themselves were not used in the statistical analysis to avoid the possibility of pseudoreplication. Data were normally distributed and satisfied the variance criteria for parametric comparisons.

## 5. Conclusions

In conclusion, we suggest four cinnamates (cinnamic acid, ρ-coumaric acid, caffeic acid, and ferulic acid) that effectively suppress the growth of *A. alternata*, the causal agent of early blight in tomato plants. The four tested cinnamates showed strong dose-dependent antifungal activity against *A. alternata*. Furthermore, foliar application of cinnamate derivatives significantly diminished the disease symptoms in infected tomato leaves, with no phytotoxicity on treated plants. Taken together, these findings suggest the potential application of cinnamate derivatives as eco-friendly control alternatives against *A. alternata* particularly and maybe other *Alternaria* species in general.

## Figures and Tables

**Table 1:** The half-maximal inhibitory concentration (IC_50_) and IC_99_ values (mM) of four tested cinnamates and the commercial fungicide Score, 25% (difenoconazole 25 EC) against *A. alternata* (*n* = *6*).

Compound	IC_50_ (mM)	95% Confidence Interval		IC_99_ (mM)	95% Confidence Interval		Overall Model Fit		
		Lower	Upper		Lower	Upper	χ^2^	Cox and Snell R^2^	*p*-Value
Cinnamic acid	0.23	0.16	0.30	2.53	1.47	7.23	1482.13	0.2565	<0.0001
*ρ*-Coumaric acid	0.15	0.13	0.17	30.68	19.96	52.14	350.12	0.0676	<0.0001
Caffeic acid	0.40	0.34	0.46	3.08	2.10	5.62	1742.86	0.9989	<0.0001
Ferulic acid	0.06	0.05	0.07	5.32	4.66	6.07	389.75	0.0750	<0.0001
Fungicide	0.05	0.05	0.06	0.79	0.71	0.88	629.70	0.1183	<0.0001

## Data Availability

All data generated or analyzed during this study are included in this published article.
